# Views upon the Statics of the Human Chest, Animal Heat, and Determinations of Blood to the Head

**Published:** 1843-04-01

**Authors:** 


### Views upon the Statics of the Human Chest, Animal Heat, and Determinations of Blood to the Head. By *Julius Jeffreys*, F.R.S. &c. &c. London, 1843.

The author has divided this work into three Parts. The first Part, to which for want of space we must confine our notice in the present number of the Journal, treats of the capacity of the chest, and the nature, condition, and office of its gaseous contents, under the term *Statics*. For these points the author claims an importance hitherto exclusively attached to the act and air of respiration, and accordingly demands a considerable modification of our previous opinions. The first point he sets about determining is the capacity of the chest. "Without," he says, "the aid of any nice apparatus to determine the quantities concerned, every person may, and often does, unconsciously make the following experiment. At the moment when he has completed an act of inspiration, and just before he performs the act of expiration, he may, instead of the latter act, force himself to continue to inspire air, when he will find that his chest can take in, before it is distended, a quantity of air very much larger than that of any ordinary breath.

"Again, after an act of expiration, at the moment when he would instinctively inspire, he may, instead of drawing in breath, continue to breath out for a great length of time, if he is in health. If it has not occurred to him to make the experiment before, he will find, to his surprise, that, at that period when the instinctive desire to take in breath had led him perhaps always to suppose his chest was empty, it still contained a vast quantity of air."

It is ascertained that the mechanism of the osseous fabric and muscles surrounding the chest is such that the utmost motion they admit of is at an end before all air is compressed out of the chest; there is, therefore, still a considerable quantity of air which cannot by any effort be expelled —this is the quantity that remains in the body after death. Adding to these the air of ordinary respiration, we have four distinct quantities; first the bulk of air, which we cannot by any effort expel—this we may call—the *residual air*. Then, on the top of this, we have the large bulk which we can expel after an ordinary expiration; this we call *supplementary air* —next comes the fluctuating *air of respiration*—and next we have the occasional quantity of air, which the chest is capable of taking in with a sigh or a yawn, after an ordinary inspiration—this our author calls the *complementary air*.

Notwithstanding the importance of these several bulks of air, it is strange that it is the air of respiration alone, which is made the subject of speculation, and upon which the various theories of respiration up to the present hour are built—with respect to the bulks of these several quantities our author sets them down as follows:

| | Cub. Inches. |
|---|---|
| Residual air .. .. .. .. .. .. .. | 120 |
| Supplementary air .. .. .. .. .. .. | 130 |
| The breath.. .. .. .. .. .. .. .. | 26 |
| Complementary air .. .. .. .. .. .. | 100 |
| | 376 |

With respect to the first and second volumes of air in the above table, as they are permanently resident in the chest, they may be called by one common name, *resident air*.

The air of respiration, which is ordinarily spoken of as that which acts on the blood in the air-cells, and when breathed out, as having given out a portion of its oxygen to the blood, and received from the blood carbonic acid and watery vapour, is denied by our author the power of ever entering the air-cells or even the smaller air-tubes. He states that it has no *direct* concern in the oxydation of the blood, and that it does not receive its carbonic acid and vapour directly from the blood. Nay more, he says that its constant presence in the air-cells would be injurious to health, and probably soon fatal to life. At these conclusions he has arrived from considering the structure of the lungs, and the position which the resident air in them occupies. When the chest expands at the moment of inspiration the *resident air* recedes before the entering breath, the portion of it most distant from the breath adding itself to the resident air already in the cells, so as to fill them up as the chest expands. This entering air cannot take the place of the resident air and descend into the cells; there is no gravitating force sufficient to make it do so; the breath at each entry can only mix itself with the uppermost portions of the resident air immediately contiguous to it. An indraught of breath of 26 cubic inches may mix itself during the respiring act which brought it in with 60 or 80 cubic inches of the resident air. Hence when a corresponding quantity of air is breathed out, it is not that which went in, but a mixture of that air with the uppermost resident air. A very natural question here suggests itself, viz. how are the purposes of respiration fulfilled, and how are the smaller air-tubes and cells supplied with air sufficiently oxygenated? Two ways offer themselves for this purpose; the first and chief is that of a progressive intermixture, proceeding by steps from without inwards at each inspiration, by which means oxygenated air is carried onwards, step by step, to the air-cells. The other way by which this object is aided is that of the expansion of a gas out of its own mixture into any contiguous volume of air, in which that gas is deficient—a process of the nature of endosmosis. Thus oxygen may travel down, and carbonic acid up, although the other gases in which they are do not change places. Thus, then, the air of respiration performs no direct duty in connexion with the blood. In its fresh state it does not come even near the the cells; its duty is altogether indirect; its action is to ventilate the chest gradually, from above downwards, and to receive the impurities gradually brought up from below, exchanged for an equal bulk of more recent air conveyed from above. Thus, then, it would appear that the *resident* air forms the chief bulk of the contents of the chest, and is that which is wholly concerned in the oxydation of the blood. The author supports his views on this part of his subject with some very ingenious arguments.

"We now surely," says our author, "may perceive the beauty of the arrangement which lodges a large quantity of air durably resident in the cells; which is there during expiration as well as inspiration, and also at the interval between the two; and is, therefore, carrying on its barter with the blood, at all times, continually and uninterruptedly.

"While this is going on, the process of *respiration* is alternately pumping in fresh air, and pumping out a mixed air: thus ventilating the chest from above, and keeping the resident air of some standard quality. Experiment tells us, this standard contains upwards of eight per cent. of carbonic acid. The quantity is probably not under ten per cent.

"In order that the action upon the blood should proceed equally and uninterruptedly, we now see the necessity of the *resident air* being larger in quantity, and the fluctuating air, the breath, much less. The case may, to a certain extent, be compared to superior double bellows, such as those of an organ, the larger portion of which is kept in a steady degree of fulness by the quickly pumping movement of the other. The bellows-man knows well that, in order to have a very steady blast, he must keep a large quantity of resident air always in his bellows."

The author puts questions which nobody else ever thought of proposing. He says, how can we do such violence to convictions in favour of fresh air, derived, not only from preconceived notions, but from our sensations also, as to believe that no better air visits the cells of the lungs, than such as is charged with carbonic acid to the extent of 8 or 10 per cent.? And can we think such air sufficiently oxygenous for carrying on the active transactions with the blood which we perceive to take place? We shall endeavour to answer these questions as far as is possible within the limits of a review, and in the author's own words:

"Since the ventilation of the innermost parts, the air-cells of the lungs does not take place, out-and-out at each act of respiration, but gradually and indirectly, through a change progressively wrought on the bulky *resident air*—since the fresh air, in short, forms so small a part of the whole, and can mix only with the uppermost portions of the resident air—since such are the conditions of the case, is it not all the more desirable that what does enter should be very fresh? Hence the jealous sensibility of the orifice of the mouth and nostrils, especially of the latter and of the larynx, to impurity of air. Hence to these portals to the lungs the care is confided, by investing them with a watchful sensibility, of seeking the purest air that can be had for the ventilating process. But as we go deeper, we find, as a matter of fact, that this sensibility decreases gradually. In the windpipe it is less than in the larynx, and in the bronchi it is less still."

In this decrease of sensibility the author perceives a corroboration of his views.

"The *statistical fact itself* is a truth too absolute to admit of corroboration. Thus, Nature having so arranged matters, that of the whole air (the resident, and the tidal or respired), that which is at the summit of the lungs shall be quite pure; and that the air shall gradually decrease in purity, until that which is in the cells is charged with 8 or even 10 per cent. of carbonic acid: such being the natural arrangement, the successive surfaces are given a corresponding degree of sensibility. Thus, we are very comfortable while we have our air-cells occupied with air so highly carbonated; it occasions no uneasiness though occupying the greater part of the lungs; but were similarly impure air to be uninterruptedly present at our nostrils during inspiration and expiration, we should turn from it with feelings of suffocation."

Our author suggests as well as answers a question which may be put here; viz. why should such impure air be always resident in the lungs, and how can we entrust to it the active duty of oxydating and defecating the blood? (The resident air in the chest he supposes to contain about 10 per cent. of oxygen.)

He considers that the state of oxygenation of the air contained in different parts of the chest is accurately proportioned to the degree of tenuity of the pulmonary membrane in contact with it; that is, in the uppermost parts of the chest, where this lining membrane is comparatively tough and thick, the air is more highly oxygenized than in the lower parts, where, if the air contained the same proportion of oxygen, it might act with too much intensity on the delicate and thin membrane lining the minute air-cells.

"The truth evidently is this, that since the air of respiration, as such, penetrates but a small way, and since it can only work its way down to the deeper part of the lungs by repeatedly mixing with successive portions of the bulky resident air, any irritating properties it possesses must become greatly diminished by the repeated dilution of it, before any portion of it can reach the cells. We may satisfy ourselves experimentally of the truth of this, by breathing out to the utmost, so as to expel as much of the resident air as we can, namely, its supplementary portion, and then, drawing in a dusty, or otherwise irritating atmosphere, we shall be immediately distressed by it, though we might have been performing ordinary respiration in it without difficulty."

The next point which our author considers is, the particular region which the air of respiration occupies in the range of the chest's capacity. We have already seen that, in the act of respiration, the breath flows in and out on the top of a large quantity of air (resident air), and within the limits of a large range yet remaining, called the complementary space, He now considers why the region of respiration is placed, as it were, two-thirds of the way up the chest, and whether it occupies the same locality in all persons, or the same in the same person at different periods. We have already seen the object of having a supply of resident air in the chest was to guard the delicate structure of the air-cells from air of too irritating a quality. This affords a reason why we do not respire as it were at the bottom of our chests, upon empty lungs. The reason why we do not respire at the summit of our chests, with the lungs already filled with the complementary as well as the resident air, must be sought in the fluctuating demand for air of respiration.

"During exercise, and especially during considerable exertion, we know that the hurried circulation of the blood through the lungs calls for a more copious supply of air. To command a range for a deeper respiration, we must either breathe out some of the resident air, and add the room thus gained to the previous range of the respiration; or, retaining in our chests the same quantity of resident air, we must increase the respiratory range by intruding upon the complemental space."

"The 'being in breath,' and its opposite 'not in breath,' appear mainly to depend on these different modes of increasing our respiration. An unpractised runner, for instance, tries to relieve himself by the former method; but he soon feels the consequence of letting out too much of his resident air, and drawing in too deeply atmospheric air, fully oxygenous, and perhaps also cold. He gets out of breath—that is, when he wants more air than usual, he cannot take in so much; a kind of asthmatic spasm prevents him from getting air enough down. .......... On the other hand, by practice he instinctively learns to keep adding air to that already present, and to breathe nearer to the top of his chest. He can then respire deeply without drawing in the fresh air too suddenly and too far into the lungs."

For the other remaining articles in this book we must refer our readers to our next number. The truly original views on so interesting and vital a subject must recommend Mr. Jeffries' work to every medical practitioner.

